# Antimicrobial Consumption and Resistance Dynamics Across Healthcare Level: Global Evidence and Stewardship Implications

**DOI:** 10.3390/pathogens15040414

**Published:** 2026-04-10

**Authors:** Neha Raut, Anis A. Chaudhary, Harshad Patil, Supriya Shidhaye, Ruchi Khobragade, Milind Umekar, Mohamed A. M. Ali, Rashmi Trivedi

**Affiliations:** 1 Department of Quality Assurance, Smt. Kishoritai Bhoyar College of Pharmacy, Kamptee, Nagpur 441002, Maharashtra, India; rautneha123@gmail.com (N.R.); harshadpatil5065@gmail.com (H.P.); shidhayesupriya@gmail.com (S.S.); ruchirakeshk@gmail.com (R.K.); 2 Department of Biology, College of Science, Imam Mohammad Ibn Saud Islamic University (IMSIU), Riyadh 11623, Saudi Arabia; aachaudhary@imamu.edu.sa (A.A.C.); mamzaid@imamu.edu.sa (M.A.M.A.); 3 Department of Pharmaceutics, Smt. Kishoritai Bhoyar College of Pharmacy, Kamptee, Nagpur 441002, Maharashtra, India; drmilindumekar@gmail.com

**Keywords:** antimicrobial resistance, antimicrobial use, One Health, antimicrobial stewardship, drug utilization, healthcare settings, vulnerable populations, safety, monitoring

## Abstract

Background/Objectives: Antimicrobial resistance (AMR) is a critical global public health challenge driven by inappropriate and excessive antimicrobial use (AMU) across human, animal, and environmental sectors. Method: This narrative review synthesizes recent evidence on antimicrobial utilization and resistance patterns. A structured search of PubMed, Scopus, and Web of Science was conducted for studies published between 2015 and 2025. Eligible sources included surveillance reports, registry-based analyses, and clinical studies. Data were qualitatively analyzed to identify key trends and regional variations. Result: Marked geographical variation in AMR was observed. Carbapenem resistance in *Escherichia coli* remains low globally (2–3%) but is higher in Southeast Asia (17–18%) and India (~40%). *Klebsiella pneumoniae* shows consistently high resistance (>40% globally; ~54% in India), while *Pseudomonas aeruginosa* exhibits stable resistance levels (35–45%). Resistance prevalence increases from primary to tertiary care settings, reflecting greater antimicrobial exposure. Vulnerable populations—including pediatric, elderly, pregnant, and immunocompromised patients—face higher risks of antimicrobial exposure and adverse outcomes, including nephrotoxicity, hepatotoxicity, and microbiome disruption. WHO AWaRe data indicate a global shift toward increased use of Watch-category antibiotics. Stewardship interventions, such as audit and feedback, prescribing restrictions, rapid diagnostics, and decision support systems, effectively reduce inappropriate AMU. Conclusions: Integrated, data-driven antimicrobial stewardship and robust surveillance systems are essential to mitigate the global burden of AMR.

## 1. Introduction

Antimicrobial resistance (AMR) occurs when microorganisms—including bacteria, viruses, fungi, and parasites—evolve mechanisms that reduce the effectiveness of antimicrobial agents, leading to persistent infections, increased transmission, and higher morbidity and mortality [1]. It is widely recognized as one of the most significant global public health challenges of the 21st century, with substantial clinical, economic, and societal consequences [2,3]. Despite ongoing global initiatives, the burden of AMR continues to rise, particularly in low- and middle-income countries (LMICs), where surveillance capacity, diagnostic infrastructure, and regulatory enforcement are often limited [4,5].

**Figure 1:**
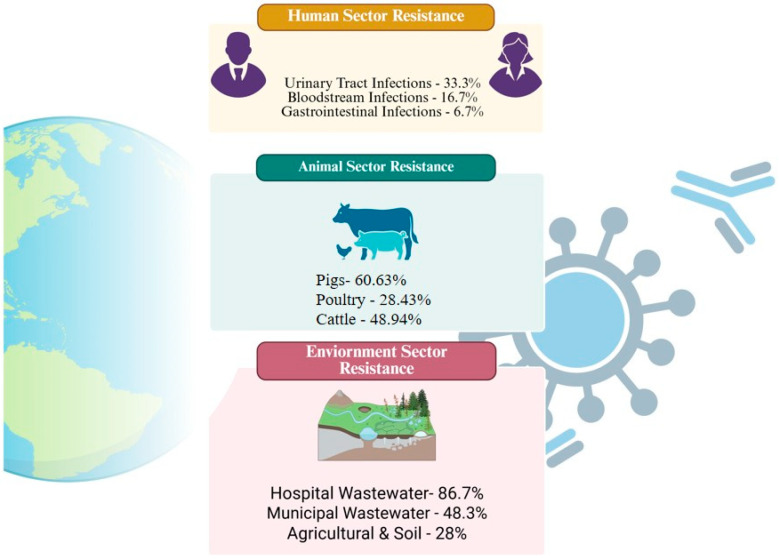
Different Sectors of Antimicrobial Burden (created by BioRender).

A key driver of AMR is the inappropriate and excessive use of antimicrobials across human healthcare, animal health, and environmental sectors as shown in Figure 1. However, treatment failure is not solely attributable to microbial resistance. As highlighted in recent studies, antibiotic failure may also result from factors such as biofilm formation, host immune dysfunction, and pharmacokinetic variability [6,7]. While this distinction is clinically relevant, it remains underexplored in the context of large-scale antimicrobial utilization and stewardship strategies. In addition to resistance, antimicrobial exposure is associated with important safety concerns, including nephrotoxicity, hepatotoxicity, and microbiome disruption, which may further complicate patient outcomes and limit therapeutic options [8]. These risks are particularly pronounced in vulnerable populations, such as pediatric, elderly, pregnant, and immunocompromised patients, who often experience higher rates of antimicrobial exposure and adverse effects.

Although numerous studies have examined AMR trends and antimicrobial consumption independently, there remains a lack of integrated, cross-sectoral synthesis linking antimicrobial utilization patterns, resistance dynamics, safety outcomes, and stewardship interventions across different healthcare levels and populations [9,10]. Furthermore, existing evidence is often fragmented across regions, healthcare settings, and data sources, limiting its applicability for global policy and practice.

Therefore, the aim of this narrative review is to synthesize current evidence on antimicrobial utilization patterns, resistance trends, safety concerns, and stewardship strategies across diverse healthcare settings and populations. This review adopts a One Health perspective to provide a comprehensive understanding of how antimicrobial use influences resistance development and to identify key gaps and priorities for optimizing antimicrobial stewardship globally.

### Methods

This study was conducted as a narrative review to summarize current evidence on antimicrobial utilization, resistance patterns, and stewardship strategies. It does not follow a formal systematic review methodology (e.g., PRISMA); however, a structured and transparent literature search approach was adopted to ensure comprehensive coverage of relevant evidence [11]. A literature search was conducted across multiple electronic databases, including PubMed, Scopus, Web of Science, and Google Scholar, to identify global, multidisciplinary studies. The search covered publications from May 2014 to December 2025. Search terms included combinations of the following keywords: “antimicrobial utilization,” “antimicrobial resistance,” “antimicrobial stewardship,” “AWaRe classification,” “One Health,” and “antibiotic consumption” [12].

Eligible sources included peer-reviewed original research articles, systematic reviews, surveillance reports, and global monitoring datasets. Studies were included if they reported data on antimicrobial use, resistance patterns, prescribing practices, or stewardship interventions in human healthcare settings (primary, secondary, or tertiary care) [13]. Studies focusing on vulnerable populations, including pediatric, elderly, pregnant, and immunocompromised patients, were also prioritized. Articles not available in English, lacking relevant outcome data, or focused exclusively on non-healthcare settings were excluded [14].

Qualitative synthesis of the relevant data was used to determine the worldwide and local trends in the utilization of antimicrobials, the prevalence of resistance, and gaps in the implementation of stewardship activities in medical care systems and the One Health domains [15]. Due to the variety of study designs and outcome measures, a narrative synthesis methodology was applied to synthesize the outcomes and highlight the emerging trends and policy implications [16].

## 2. Global Patterns of Human Antimicrobial Exposure

### 2.1. Antimicrobial Exposure Across Countries

Global evidence demonstrates substantial regional variation in antimicrobial resistance, with the highest burden observed in LMICs, particularly in Sub-Saharan Africa and Southeast Asia. While high-income regions such as Europe and the Americas benefit from established surveillance systems and relatively stable resistance trends, LMICs experience disproportionately higher resistance rates. This disparity is primarily driven by limited diagnostic infrastructure, weak regulatory enforcement, and widespread empirical antibiotic use. Furthermore, higher infectious disease burden in these regions amplifies antimicrobial exposure, accelerating resistance development.

Low- and Middle-income countries, which face the largest and most prevalent communicable disease burden and the least available resources, have minimal information on the epidemiology and strains of antimicrobial resistance (AMR) [17]. The least developed coverage is in Sub-Saharan and South and Southeast Asia, in contrast to the high earnings and developed countries such as the United States and European countries [18]. In low-income settings, the obstacles are enormous due to weak laboratory and messaging infrastructures, limited resources, the absence of trained and qualified staff, and various socioeconomic and behavioral drivers of resistance [19].

The strain of AMR in the WHO Region of the Americas for pathogens and drugs. Analysis discloses that 11.1% of the total calculated worldwide deaths attributable to AMR and 11.5% of the worldwide deaths associated with AMR happened within the Americas. We identified substantial strain generated by *S. aureus* and *E. coli*, with each responsible for more than 100,000 deaths associated with AMR in the region [2]. The Americas have a long history of monitoring of AMR, but there are still obstacles to translating this monitoring into public action [20].

Antimicrobial consumption has shown regional variation across the WHO regions. Increased consumption has been reported in the WHO Southeast Asia Region, African Region, Region of the Americas, and Eastern Mediterranean Region, whereas a decreasing trend has been observed in most countries in the WHO European Region and Western Pacific Region. These findings highlight geographic disparities in antimicrobial use and emphasize the need for region-specific antimicrobial stewardship strategies [21].

In the WHO European region, 0.9 million deaths were caused by both open and exposed and resistant bacterial agents to approximate the AMR strain [22]. Seven pathogens were each responsible for deaths associated with AMR in the WHO European region: *Escherichia coli*, *Staphylococcus aureus*, *Klebsiella pneumoniae*, *Pseudomonas aeruginosa*, *Enterococcus faecium*, *S pneumoniae*, and *Acinetobacter baumannii* [23]. Sub-Saharan Africa (SSA) had the highest mortality rate (23.5 deaths per 100,000) attributable to AMR compared to other regions. The Sub-Saharan Africa (SSA) region is disproportionately affected by AMR, in part owing to the prevailing high levels of poverty, which strain health systems, lead to poor regulation of antimicrobial use, and leave few options for ineffective antimicrobials [24]. Sub-Saharan Africa (SSA) is encountering a rise in AMR and its harmful impacts. Our review shows a prevalence of MRSA and ESBL-producing *Enterobacterales* of around 40% in sub-Saharan Africa (SSA), which are globally recognised as priority-resistant germs. There was furthermore a high prevalence of fluconazole-resistant candidiasis and cryptococcosis, conditions that primarily influence people living with HIV, who are multiple in Sub-Saharan Africa (SSA). Finally, an increase in resistance was detected in infections triggered by *Shigella*, *Salmonella* spp., and *V. cholerae*, leading to Diarrhoeal diseases and high mortality [25].

It is interesting to note that carbapenems, a class of antibiotics including doripenem, ertapenem, meropenem, and imipenem, have a significant impact on the treatment of multidrug-resistant infections and hospital-acquired extended-spectrum β-lactamase infections. However, the use of carbapenem antibiotics worldwide is risky due to the evolution of resistance to them [26,27].

**Figure 2:**
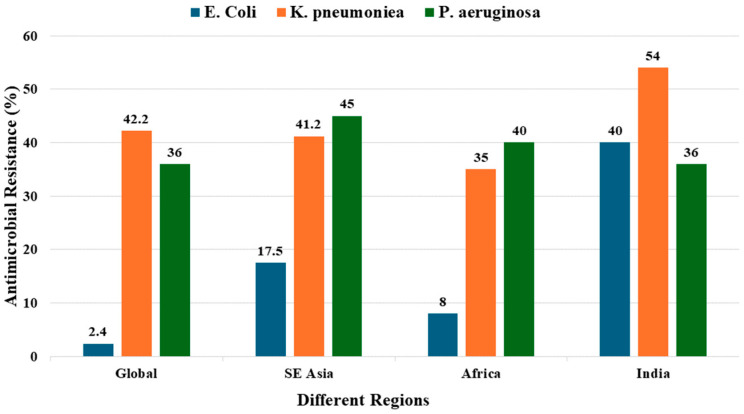
Worldwide Resistance to Carbapenems.

Carbapenem-resistant organisms represent a watershed signal of treatment-limiting resistance, as carbapenems serve as last-resort antibiotics for numerous infections. Global monitoring data from 2023 reported remarkably low *E. coli* carbapenem resistance at 2.4%, but this masks dramatic local variation, with Southeast Asia reporting 17.5% and India reporting 40% [28]. *Klebsiella pneumoniae* carbapenem resistance showed comparable trends. Global resistance reached 41.2% according to GLASS 2025 data, but India documented 54% in 2023, up from 41% in 2017. *Pseudomonas aeruginosa* carbapenem resistance remained relatively stable at 36% globally and in India, though Southeast Asian rates reached 45% [29]. Carbapenem resistance globally and in different countries is given in Figure 2 [30,31,32].

The COVID-19 pandemic further influenced global antimicrobial consumption patterns. During the pandemic, increased empirical antibiotic prescribing was widely reported due to concerns regarding secondary bacterial infections, limited diagnostic capacity, and clinical uncertainty. Comparisons between pre- and post-COVID-19 periods indicate a notable rise in antimicrobial use during 2020–2021, followed by partial stabilization or decline in some regions; however, overall consumption remained elevated in several low- and middle-income countries. These changes may have contributed to the acceleration of antimicrobial resistance, highlighting the importance of strengthening antimicrobial stewardship strategies in the post-pandemic period.

### 2.2. Antimicrobial Utilization and Resistance in Low- and Middle-Income Countries

Antimicrobial resistance is a disproportionately serious problem in low- and middle-income nations (LMICs), notably in Southeast Asia and India, where antimicrobial use is inappropriate, extreme, and excessive in both medical care and community settings. Rapid population increase, heavy strain of infectious diseases, poor admittance to diagnostics, poor management, and regulatory oversight are some of the aspects that have led to the thorough empirical and irrational antibiotic prescribing behaviors [33,34].

Population-level analyses have shown a notable increase in antimicrobial use in LMICs over the past 20 years. There has been an upsurge in the total use of antibiotics by approximately 65% throughout the world, incorporating fast development in India, China, and other Southeast Asian nations, chiefly due to increased availability and uncontrolled application of broad-spectrum agents between 2000 and 2015 [35]. In several LMIC settings, antibiotics are persistently available over the counter without prescription, further exacerbating inappropriate application and accelerating resistance pressure [36].

According to WHO monitoring data, resistance rates have substantially increased in LMICs compared with high-income countries. *Escherichia coli* resistance to third-generation cephalosporins and fluoroquinolones exceeds 50% in several Southeast Asian countries, and resistance to carbapenems in Klebsiella pneumoniae has reached alarming levels, particularly in India [37]. These trends are further amplified in tertiary and intensive care unit settings, where antimicrobial exposure is prolonged, and infection prevention measures are frequently suboptimal [38].

The lack of appropriate antimicrobial stewardship infrastructure, microbiology capacity, and surveillance systems in LMICs impedes early detection and control of antimicrobial resistance. Moreover, the absence of a One Health surveillance approach to connected sectors in the human, animal, and environment domains masks the actual burden of resistance transmission and dissemination [39]. Addressing AMR in LMICs thus necessitates coordinated policy interventions, strengthened regulatory frameworks, enhanced access to diagnostics, and context-specific stewardship methods customized to resource-limited settings [40]. Overall, these findings indicate that antimicrobial resistance is not uniformly distributed but is strongly influenced by healthcare infrastructure, regulatory capacity, and antimicrobial consumption patterns.

### 2.3. Exposure in Different Healthcare Settings

#### 2.3.1. Primary Care

There are themes of Patient, Provider, Healthcare systems, Intervention, Absorption, and Implementation. These themes were further subdivided into 18 sub-themes, which provide a comprehensive idea of those factors that influence the use of antimicrobials in Primary health care settings [41]. There is persuasive and convincing evidence suggesting that there are direct correlations between extreme and excessive antibiotic prescribing and application in the Primary health care setting, with increases in AMR rates in groups across the world. AMS plans in the rural and remote Primary health care settings tend to focus on condition care providers and patient education, monitoring, clinician support systems, and nationwide policy changes [42]. Multiple limitations in antibiotic prescribing within the Primary health care system. Specifically, 14% of doctors consistently prescribe antibiotics without a prescription; 21% employ injectable antibiotics; and 66.5% occasionally prescribe numerous and manifold antibiotics to treat a bacterial infection of a single localization, which is prohibited by the Standard. Only 41.2% of doctors evaluate the effectiveness of antibiotic therapy according to recommendations, within 48–72 h [43]. As a result of the study of the views of primary care physicians on the application of antimicrobial drugs in their practice, three essential conclusions were made: (1) Physicians are aware of the value of the AMR problem in PHC. (2) They are generally recognizable with the recommendations on the choice and application of antibiotics for the treatment of widespread infections. (3) Nevertheless, in practice, these recommendations are frequently violated.

#### 2.3.2. Secondary/Tertiary Hospitals

Physicians feel that it is hard and challenging to get antibiotic-naive patients, notably in a secondary and tertiary centre. So, they are compelled to employ greater antibiotics. He has furthermore observed that resistance levels are higher than before and feels that excessive antibiotic use in non-medical sectors could be playing a role [44]. One secondary-level hospital managed to document and quantify prescribed antibiotics. These measures were taken to obtain a sense of the real antibiotic application [45]. These hospitals need to encompass generalized physician and employee education efforts, institution-specific antimicrobial appropriation protection, and the advancement of multidisciplinary ASP groups, which later on involve physician, pharmacist and microbiological representatives [46].

#### 2.3.3. Telemedicine/Online Prescribing

The application of telecommunications and information technology to provide access to condition assessment, diagnosis, intervention, consultation, oversight, and information across distances [47]. Telemedicine antimicrobial stewardship has been shown to increase adherence to guidelines, reduce prescription rates, and reduce days of therapy (DOT) without substantially affecting mortality [48]. The application of remote consultations is expected to increase in primary care internationally, guaranteeing the safety and quality of these consultations, and avoiding harmful and detrimental impacts on antimicrobial resistance should be prioritized [49].

#### 2.3.4. WHO AWaRe (Access, Watch, Reserve) Classification Trends

Antibiotic consumption tracking is vital and crucial for setting goals and following up activities aimed at optimizing antibiotic application. It is because of this that the WHO has come up with the AWaRe system, which groups antibiotics into three categories: (1) Access (narrower spectrum agents with less potential resistance). (2) Watch (widening spectrophotometry and enhanced resistance). (3) Reserve (antibiotics of the last resort) [50].

**Figure 3:**
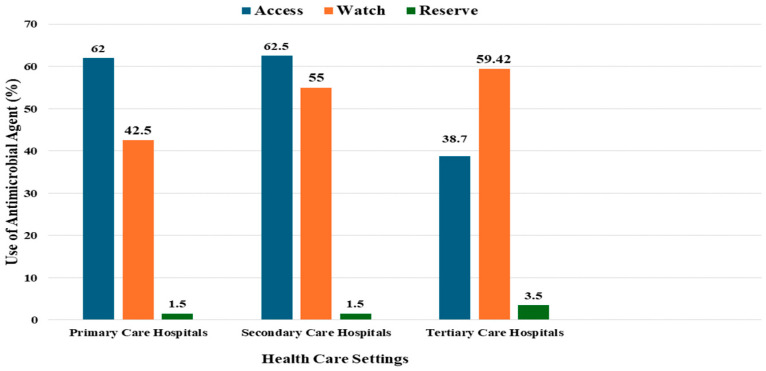
Antimicrobial Use According WHO AWaRe Classification.

The AWaRe classification scheme and its associated target are widely popular in most countries across the globe [51]. When treating a variety of common infectious diseases empirically, antibiotics in the Access category are considered first- or second-line treatments. The Watch category consists of antibiotics with a higher probability of acting on resistant selection and are selected as first- or second-line treatment only because of a restricted range of indications. These are the antibiotics that stewardship programs should monitor and prioritize. A group of last-resort antibiotics in the reserve category must be closely monitored and used only under very particular circumstances to preserve their efficacy [52]. AWaRe classification showing a notable increase in the application of Watch category antibiotics [53]. Antibiotic use according to the WHO AWaRe classification is shown in Figure 3. Antibiotics in the Access category were consumed in large numbers. There were no standard treatment guidelines in the hospital. In all, 98.0% of antibiotics were consistent with the hospital formulary and prescribed under generic names [54]. 

### 2.4. Patterns of Antibiotic Classes Used

#### 2.4.1. Broad-Spectrum vs. Narrow-Spectrum

Compared to narrow-spectrum antibiotics, which are effective against a limited number of germs, broad-spectrum antibiotics are used to treat a greater variety of bacterial illnesses. When the cause of the infection is unknown, broad-spectrum antibiotics are generally beneficial [55]. Empirical application of broad-spectrum antibiotics had been recorded. Financial crises have led to an underuse of narrow-spectrum antibiotics. The pattern of antibiotic prescriptions for hospital patients was inconsistent with current and ongoing recommendations. The rationale and target medication therapy for broad-spectrum antibiotics must be provided, and strict adherence to recommendations must be ensured. Broad-spectrum antibiotics are mostly used to improve initial conditions in severely ill intensive care unit patients. Patients’ treatment plans should be chosen based on their safety profile and propensity to resist antibiotics [56].

#### 2.4.2. OTC and Non-Prescription Antibiotic Use

Dispensing of non-prescription antibiotics frequently requires obtaining redundant and superfluous or short-course medication and inappropriate drugs and doses. The high prevalence of non-prescription antibiotic application globally warrants more standardized studies and critical and imperative measures to adjust this risky practice [57]. According to antibiotic dispensing guidelines in most of the countries, antibiotics should be dispensed only upon a valid prescription. Additionally, each prescription should be used for the related treatment of the precise and particular condition state as prescribed by the physician [58]. The highest and most prevalent percentages of prescription-free antibiotic dispensing observed during the simulated patient procedure matched those reported in Asian nations. Nonetheless, the lowest percentage of prescription-free antibiotic dispensing was noted in an Indian [59]. Antibiotic dispensing without a prescription is still common and widespread in many nations, especially those with low and intermediate incomes.

Empower these medical professionals, especially in developing nations, by implementing administrative and/or educational strategies to decrease the dispensing of antibiotics without a prescription [60].

Over-the-counter (OTC) use of antibiotics contributes to the growing burden of antimicrobial resistance. Only a few dispensers and patients were aware of AMR and explained AMR as a potential effect of inappropriate and overuse of antimicrobials through OTC [61].

## 3. Antimicrobial Exposure in Vulnerable Populations

Across all vulnerable groups, increased antimicrobial exposure, altered pharmacokinetics, and frequent healthcare contact emerge as common drivers of both resistance and adverse outcomes [62].

### 3.1. Pediatric Population

Antibiotic resistance is a public health danger of the utmost importance, notably when it comes to children [63]. In Europe, MDR infections in pediatric patients may account for up to 30% of all infections. In regions of the Middle East, 90% of newborns with sepsis, admitted in ICU, had resistant bacteria, in some areas of Southeast Asia, 83% of children have *E. coli* resistance to first line antibiotics, Antibiotic-resistant bacteria were discovered to be the cause of 66% of neonatal sepsis and meningitis in Sub-Saharan Africa, and 20% of pediatric patients receiving colistin to treat existing MDR Gram-negative bacteria became resistant [64]. The juvenile population should have access to pharmaceutical treatments that meet their needs in terms of prescribing, safety, posology, and efficacy. This is especially true when one considers the significant pediatric variability in pharmacokinetics (PK), which is somewhat age-dependent [65].

### 3.2. Elderly Population

Infections, which often present atypically and are linked to high morbidity and death, are particularly dangerous for elderly individuals. Antimicrobial therapy for elderly patients with infectious illnesses indicates a clinical challenge, placing an increasing burden on global healthcare systems [66]. Due to factors like immunosenescence, multimorbidity, and frequent and repeated medical care exposure, antibiotic resistance in the elderly is an increasing medical care concern [67].

Strategies to Combat Antibiotic Resistance in the Elderly.

- Novel Antibiotics
- Bacteriophage Therapy
- Antivirulence Therapies
- Probiotics and Faecal Microbiota Transplantation
- Vaccine Development
  - Multi-Epitope Vaccines Using Immuno-informatics and mRNA Technologies
  - Nanoparticle-Based Vaccines
  - Reverse Vaccinology and Antigen Discovery
  - Lipopeptide Adjuvants
  - Other Strategies to Enhance Immunity Following Vaccination
- Antimicrobial Stewardship Programs
- Advanced Diagnostic Techniques

**Figure 4:**
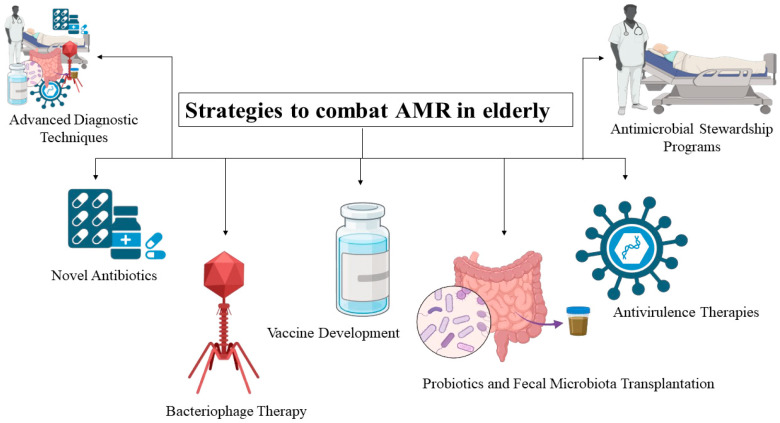
Strategies to Combat Antibiotic Resistance in the Elderly (created by BioRender).

Geriatrics have a much-increased risk of experiencing acute renal injury when compared to the wider adult population. In elderly persons, the risk of AKI is 15.1% [68]. According to Figure 4, all the strategies listed above aim to combat antibiotic resistance in the elderly population.

### 3.3. Pregnant & Postpartum Women

Due to the expanding abuse and overuse of antimicrobials, post-partum infections are a remarkable therapeutic challenge, because they are caused by an alarmingly rising rate of germs resistant to the widely utilized medications [69]. The high prevalence of AMR in women in the peri-/post-partum period in resource-limited countries could be largely clarified by the persistent misuse of antibiotics in these settings [70]. Pregnant women have an increased risk of urinary tract infections. However, using antibiotics without a culture and a sensitivity test may contribute to antimicrobial resistance [71]. Pregnant women are at increased risk of infections caused by antimicrobial-resistant pathogens, particularly those resistant to commonly used antibiotics such as ampicillin [72]. In addition, more than half of the isolates exhibited resistance to Trimethoprim-sulfamethoxazole and sensitivity to Ciprofloxacin and Nalidixic acid [73].

### 3.4. Immunocompromised Groups

Antimicrobial resistance (AMR) disproportionately influences patients who are immunocompromised (such as patients from intensive care units (ICUs), HIV-positive patients, patients with malignancies or transplant patients) due to their regular and recurring encounters with the health-care system and repeated, prolonged exposure to antibiotics [74].

HIV infection is associated with methicillin resistance in *S. aureus* [75]. In cancer patients, resistance to antibiotics, such as fluoroquinolones, aminoglycosides, and third-generation cephalosporins, was regularly observed in diverse pathogens [76]. Without adequate antimicrobials, patients cannot undergo transplant surgery safely [77]. There is also a risk of developing antimicrobial resistance.

In solid organ transplant recipients, several multidrug-resistant bacterial pathogens pose significant challenges due to their resistance profiles and the infections they cause. Methicillin-resistant Staphylococcus aureus (MRSA) commonly affects liver, lung, and heart transplant patients, causing bacteremia, pneumonia, and surgical site infections [77]. Vancomycin-resistant enterococci primarily cause bacteremia in liver and heart transplant recipients [78]. Extended-spectrum beta-lactamase (ESBL)-producing *Enterobacterales*, such as *Escherichia coli* and *Klebsiella pneumoniae*, are common in liver, heart, and kidney transplants, leading to bloodstream and intra-abdominal infections [79,80]. Carbapenemase-producing *Enterobacterales*, especially *K. pneumoniae*, are critical threats in liver, lung, and kidney transplants, with increasing resistance trends over time [81]. Multidrug-resistant or extensively drug-resistant *Pseudomonas aeruginosa* mainly affects liver and lung recipients, while carbapenem-resistant *Acinetobacter baumannii* is prevalent in abdominal organ and lung transplants [82]. These resistant infections contribute to high morbidity and mortality rates post-transplantation, underscoring the need for vigilant antimicrobial stewardship and infection control measures tailored to the type of organ transplanted [79,83].

## 4. Determinants of Antimicrobial Use

### 4.1. Healthcare System Factors

Antibiotic use decreased as a result of clinical recommendations tailored to the situation and also because of controlled over-the-counter antibiotic prescriptions. There are no rules governing the prescription of antibiotics, and doctors often rely on their own expertise and experience, which can lead to overprescription or inadequate use of antibiotics [84]. Guidelines are more effective when combined with stewardship programs and ongoing education [85].

Rational drug administration is defined as patients receiving medications appropriate to their clinical requirements, in doses that meet their individual needs, for a sufficient period of time, and at the minimum cost. Rational treatment requires the appropriate selection of an antibacterial drug, preferably based on pathogen identification by an antibiogram, bacterial resistance profiles, drug safety profiles, dosing, duration, effectiveness, and patient-specific factors. Irrational application is specified as ‘overuse, underuse or misuse of medicines’ and outcomes in wastage of scarce resources and pervasive and far-reaching condition dangers. Irrational application includes absence of public consciousness, unrestricted drug entry, inadequate medical training and advice, pharmaceutical marketing, limited diagnostic instruments, economic pressure and cultural attitudes [86].

Antibiotic prescribing in public OPDs is driven by a combination of knowledge gaps, attitudinal limitations, patient expectations, time restrictions, and setting-specific difficulties [87]. Physician autonomy can lead to variability in prescribing, with some clinicians relying on personal experience rather than guidelines, particularly senior doctors. Restrictive policies and stewardship interventions (for instance, compulsory indications, audits) can reduce inappropriate application, but may face resistance if not supported by education and evident communication [88].

### 4.2. Patient-Related Factors

Physicians’ antibiotic use is influenced by their perceptions of their patients [89]. Patients do not wish to waste money on another prescription; instead, they employ their earlier prescription to get drugs from a private or public pharmacy [90]. The acknowledged necessity for antibiotic prescriptions may have been influenced by patients’ socioeconomic characteristics, including occupation, education, and literacy [91]. Antibiotic prescriptions are influenced by the patient–physician interaction as well as deliberate exaggeration or misrepresentation of symptoms [92]. Patient attitudes, knowledge, or principles influence antibiotic prescribing by primary care physicians, for example, through misconceptions about the role of antibiotics and their effectiveness in treating viral infections and infectious diseases [93].

### 4.3. Socio-Cultural and Economic Factors

Socio-cultural and economic factors substantially affect antimicrobial use and resistance patterns across diverse regions. Cultural norms, such as social expectations and established routines around antibiotic application, can encourage overconsumption [94]. Antibiotic abuse was found to be more prevalent in people with lower socioeconomic status than in those with higher socioeconomic status [95]. Economic stresses on individuals create a fertile ground for inappropriate application. Also, sociocultural influences, comprising peer advice and conventional and customary principles, are responsible for inappropriate application [96].

### 4.4. Pharmaceutical and Supply-Chain Factors

Pharmaceutical and supply-chain factors critically affect antimicrobial use by influencing the availability, affordability, and quality of medicines. Inefficient supply chains, notably in low- and middle-income countries, frequently generate antimicrobial shortages that lead to inappropriate substitutions and increased resistance risk [97]. Strengthening supply chain management through better forecasting, procurement, regulation, and integration into antimicrobial stewardship programs is vital and crucial to ensure steady and regular entry to quality antimicrobials and combat resistance globally [98].

## 5. Safety Issues Related to Antimicrobial Exposure

### 5.1. Adverse Drug Reactions

Antimicrobials are among the leading causes of ADRs in hospitals and community care, accounting for 20% of antibiotic-treated inpatients experiencing at least one ADE and a substantial portion of ED visits and spontaneous reports [99]. Skin reactions (rashes, urticaria, serious cutaneous reactions) are the most often reported, particularly in children and in pharmacovigilance databases [100].

Cutaneous adverse drug reactions (ADRs) commonly manifest as exanthema, urticaria, and severe forms such as Stevens–Johnson syndrome (SJS) or toxic epidermal necrolysis (TEN). These reactions are frequently associated with drug classes including penicillins, cephalosporins, sulfonamides, and various non-β-lactam antibiotics [101]. Neurologic ADRs can present as confusion, seizures, and other central nervous system effects, often linked to fluoroquinolones and high doses of β-lactams among other drugs [102]. Gastrointestinal and flora-related ADRs typically include diarrhoea and Clostridioides difficile infections, which are commonly caused by broad-spectrum antibiotics and fluoroquinolones [103]. Recognizing these typical manifestations alongside their notable drug classes is crucial for early diagnosis and management to reduce morbidity associated with ADRs [104]. Overall, antimicrobial agents are among the most frequently implicated drug classes in these adverse reactions across different organ systems [105].

One of the most common causes of acute kidney damage (AKI) among hospitalized patients is still medication-induced nephrotoxicity. Antibiotics and other antimicrobials are recognized triggers of both structural and functional renal impairment within the broad spectrum of drugs linked to AKI [106]. The spectrum of antimicrobial-induced kidney toxicity extends from minor forms of tubular damage to severe tubular necrosis and cellular demise [107]. Mechanisms of antibiotic-induced nephrotoxicity contain glomerular injury, tubular injury or dysfunction, distal tubular blockage by casts, and acute interstitial nephritis mediated by a type IV hypersensitivity reaction. Clinical symptoms of antibiotic-induced nephrotoxicity include acute tubular necrosis, acute interstitial nephritis, and Fanconi syndrome [108]. High-risk agents contain aminoglycosides, vancomycin, polymyxins, amphotericin B, and some β-lactams responsible for nephrotoxicity [109]. Newer antibiotics since 2018 generally show low reported nephrotoxicity, but monitoring is limited [110].

A myriad of therapeutic medications used in clinical practice have resulted in hepatotoxicity, with more than 1100 pharmaceutical and chemical products associated with liver impairment. Drug-induced liver damage is responsible for over 40,000 fatalities annually in 17 countries and accounts for 7% of adverse responses, 2% of jaundice, 1% of acute liver failure, and more severe hepatitis [111]. When cell necrosis and apoptosis outpace the liver’s capacity to repair, hepatic damage becomes apparent [112]. The toxins target liver mitochondria by increasing reactive oxygen species, which then react with sulfhydryl groups and induce lipid peroxidation. Furthermore, cytochrome P450-2E1 (CYP2E1), a cytochrome P450 (CYP450) isoenzyme that catalyzes various compounds for biotransformation, including small organic molecules, pharmaceutical drugs, toxic chemicals, and environmental pollutants, is an important and significant oxidative free radical inducer in the cell that damages the liver and causes complete liver dysfunction [113].

Many antibiotic reactions recorded as allergies were unknown or not remembered by the patient, cutaneous reactions unconnected to drug hypersensitivity, drug-infection interactions, or drug intolerances [114]. Adverse reactions to non-beta-lactam antimicrobials are relatively regular and recurring [115]. Allergy cross-reactivity is mostly caused by the β-lactam ring’s R-group side chain [116]. Cefazolin is unusual in that it lacks a comparable R-group side chain to other β-lactams, thereby enabling its extended applicability [117]. Several studies have shown an association between early antibiotic use and the development of allergic diseases. Patients with allergic diseases, such as asthma, have an increased risk of being prescribed antibiotics for infections compared to the broader population [118].

### 5.2. Microbiome Disruption

**Figure 5:**
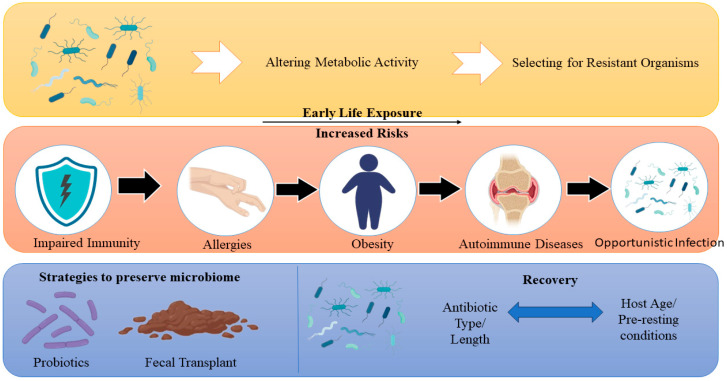
Microbiome disruption (created by BioRender).

Antimicrobial use disrupts the gut microbiome by decreasing species diversity, altering metabolic activity, and selecting for resistant organisms, leading to gut dysbiosis with both short- and long-term clinical outcomes [119]. This is well illustrated schematically in Figure 5. This interruption impairs immune system development and function, rising risks of infections, autoimmune diseases, and metabolic disorders such as obesity and allergies, particularly when antibiotic exposure occurs early in life [120]. The microbiome’s recovery after antibiotic treatment varies depending on factors such as antibiotic type, duration, host age, and pre-existing conditions, with some changes persisting for years and complete restoration often incomplete [121]. Antibiotic-induced dysbiosis also supports the overgrowth of opportunistic bacteria and the accumulation of resistance genes, complicating infection control and potentially worsening clinical outcomes [122]. Understanding microbiome dynamics during and after antibiotic exposure is vital for building techniques that preserve microbiome function and reduce long-term metabolic and immune-related outcomes [123]. Figure 5 gives an overview of microbiome disruption due to antimicrobial use.

## 6. Evidence from Population-Level and Registry-Based Data

### 6.1. National Antimicrobial Consumption Data Systems

The European Centre for Disease Prevention and Control (ECDC) oversees the European Surveillance of Antimicrobial usage Network (ESAC-Net), which gathers and analyses data on antibiotic use from 30 EU/EEA countries [124]. The European Antimicrobial Resistance Surveillance Network (EARS-Net) and the European Surveillance of Antimicrobial Consumption Network (ESAC-NET) gather population-level data over time and across the European Economic Area (EEA). While ESAC-NET measures the use of systemic antibiotics, EARS-NET assesses antibiotic resistance among invasive isolates of sentinel bacteria. Together, data from these networks were interrogated to delineate correlations between antimicrobial consumption and resistance [125]. Analysis of ESAC-NET and EARS-NET data indicates robust relationships between total antimicrobial intake and the prevalence of essential resistance phenotypes in sentinel germs, with spatiotemporal distributions varying. In conclusion, there is a clear correlation between antimicrobial resistance and the use of certain medicines, including carbapenems, macrolides, nitroimidazoles, fluoroquinolones, extended-spectrum penicillin/β-lactamase inhibitor combos, and second- and third-generation cephalosporins [126]. Patterns of antimicrobial consumption can be deeply disrupted during unforeseen and unanticipated situations, such as pandemics [127].

In its yearly reports, the World Health Organization’s (WHO) Global Antimicrobial Resistance and Use Surveillance (GLASS) program included data on antimicrobial consumption (AMC) and antimicrobial resistance (AMR) [37]. The GLASS document demonstrated a statistically significant beneficial linear relationship between extended-spectrum cephalosporins (ceftriaxone and ceftazidime) and fluoroquinolones (ciprofloxacin and levofloxacin) in the resistance of *E. coli* and *Klebsiella pneumoniae* [128].

Active substances that currently have an active ingredient code are eligible for the Defined Daily Dose (DDD). It is the anticipated and normal daily maintenance dosage of an antimicrobial medication or medications used for their primary indication in humans. The ATC/DDD strategy was developed to optimize patient care by monitoring antibiotic usage and conducting a study [129]. As part of the larger strategy of antimicrobial stewardship (AMS) initiatives for optimizing antimicrobial therapy, DDD is a well-recognized indicator on a global scale and a useful and advantageous step in assessing total drug consumption [130]. DDD most likely overestimates the use of some antibiotics, including beta-lactams, particularly in settings such as intensive care units [131]. A precise and accurate picture of antibiotic use cannot be obtained from a single statistic, such as Days of Therapy (DOT) or Defined Daily Dose (DDD). Hospitals should apply DDD or DOT in combination with Days of antibiotic spectrum coverage (DASC) or Antibiotic spectrum index (ASI) to assess antibiotic consumption and the efficiency of AMS programs [132].

### 6.2. Strengths and Limitations of Registry-Based Research

Large scale and representativeness: National or local monitoring and administrative datasets allow population-level estimates and cross-country comparisons of antimicrobial consumption and resistance, as in WHO-GLASS AMC/AMR analyses and worldwide structured and orderly assessments [133]. Longitudinal trend analysis: Multi-year hospital or local datasets allow monitoring AMU and AMR trends and associations over time [134]. Hypothesis generation and policy direction: Linked datasets (for example, antibiotic application + AMR + population attributes) provide added value for policy and guideline development and for optimizing monitoring design [135]. Feasibility and low minimal and negligible cost: Routine microbiology and administrative data are frequently the sole sustainable choices in LMICs [136].

Ecological bias and confounding: Many registry-based and linkage studies are ecological, making causal inference hard, challenging and vulnerable to unmeasured confounding [137]. Data completeness and bias: Underuse of microbiology, missing denominator data, incomplete coverage of primary care, and heterogeneous methods limit accuracy and comparability, especially in LMICs [138]. Heterogeneous definitions/techniques: Differences in ATC/DDD application, case definitions, and monitoring protocols complicate between-country and between-sector comparisons [139]. Limited patient-level detail: Many registries lack clinical seriousness, indication, or comorbidity data, limiting alteration and interpretation [137].

### 6.3. Examples of Registry-Based Findings

Regional human AMC in Tanzania showed varying DID (36.7–50.2), incorporating a 29% reduction followed by increases, demonstrating how downstream facility-level data can quantify changing application over time [140]. GLASS 2022 worldwide AMC: Several countries with strong and durable stewardship programs (for instance, the UK, the US, and Sweden) demonstrate simultaneous decreases in AMC and AMR over time. A scoping review of primary-care AMR data sources highlights that community AMU/AMR monitoring is significantly less developed than hospital monitoring and frequently lacks integrated, real-time registries. The GLASS 2022 analysis shows that countries with robust AMS and declining AMC exhibit lower AMR rates, supporting stewardship’s impact at the national scale [14].

## 7. One Health Interactions Affecting Human AMU

Antimicrobial resistance (AMR) is a global threat, driven by interconnected factors across human, animal, and environmental domains, a concept central to the One Health approach [141]. Animal antimicrobial use (AMU) contributes to human exposure through direct contact, food consumption, and environmental contamination, while environmental reservoirs (water, soil, and the food chain) further intensify the spread of resistance genes [142,143]. Integrated monitoring systems and trilateral datasets are necessary and fundamental for monitoring AMU and AMR across sectors, but notable gaps persist in worldwide One Health tracking, particularly in low- and middle-income countries [143].

### 7.1. Animal Antimicrobial Use Contributing to Human Exposure

The application of antimicrobials in food-producing animals is an important and significant driver of AMR, with resistant bacteria and genes communicated to humans via direct contact, the food chain, and environmental routes [144]. Mass medication and in-feed application of critically significant and essential antimicrobials in animals (for example, cephalosporins, fluoroquinolones, colistin) are of specific and precise concern for human condition [145]. Recent studies show bidirectional effects of AMU on AMR in both human and animal bacteria, particularly for zoonotic germs [146].

### 7.2. Environmental Contamination (Water, Soil, Food Chain)

Antibiotic residues and resistant bacteria enter the environment through wastewater, agricultural runoff, and improper disposal, contaminating water, soil, and food [147]. Environmental compartments act as reservoirs, enabling the horizontal transfer of resistance genes and amplifying AMR spread [148]. Contaminated crops and animal products serve as supplementary pathways for human exposure to AMR [149].

### 7.3. Interventions and Stewardship Programs

#### 7.3.1. Hospital Antimicrobial Stewardship Programs (ASP)

**(Audit & feedback, Restrictive policies, Rapid diagnostics)**

A pre/post-study in an Italian tertiary hospital showed that bi-weekly audits and feedback led to a 21% reduction in antimicrobial consumption, notable cost savings, and enhanced prescription quality without affecting patient outcomes [150]. A meta-analysis of 52 studies found that ASPs (including audit/feedback) reduced antibiotic prescriptions by 10% and overall consumption by 28% across both high- and low-income settings [151]. Audit and feedback, real-time recommendations, and enablement were among the most effective AMS intervention functions for changing prescriber behavior in hospitals [152]. Restrictive policies (for example, requiring specialist consent for specific antibiotics) were effective in decreasing inappropriate application and resistance rates in LMIC hospitals, though obstacles include the absence of leadership and resources [153]. International specialists suggest limiting prophylactic, redundant, and superfluous antibiotic application in hospitals, with guidelines stressing short perioperative regimens and adherence tracking [154]. Rapid diagnostic tools, when integrated into stewardship programs, improve targeted therapy and reduce broad-spectrum antibiotic application [155]. Both human and veterinary sectors identified diagnostics (comprising fast tests) as fundamental to evidence-based prescribing and ongoing AMR monitoring [156].

#### 7.3.2. Community and Primary Healthcare Interventions

**(Clinical decision support systems, Delayed prescribing strategies)**

Digital instructional and informative interventions and Clinical decision support systems in ASPs enhanced knowledge, prescribing behavior, and were flexible to varied settings, though long-term impact data are limited [157]. Australia’s National Centre for Antimicrobial Stewardship utilizes data science and CDSS for audit/feedback in both primary care and veterinary clinics [158]. Community Health Workers and animal condition workers play essential functions in condition promotion, education, and delayed prescribing plans, notably in Africa, but need standardized functions and sustainable funding [159]. Community-based interventions, comprising delayed prescribing and education, enhanced knowledge and reduced inappropriate antimicrobial application [160]. There are other digital tools available, which are mentioned in Table 1.

### 7.4. Role of Digital Health and Artificial Intelligence in Antimicrobial Stewardship

Digital medical care technology and Artificial Intelligence (AI) have been increasingly recognized as powerful levers in enhancing Antimicrobial Stewardship (AMS) programs. Digital Health Records (EHRs), Clinical Decision Support Systems (CDSSs), E-prescribing Systems, and Real-Time Surveillance Systems have been found to encourage the timely and data-driven rationalization of antimicrobial prescribing approaches with immediate feedback to medical care professionals [161].

Evidence suggests a noticeable reduction in inappropriate antimicrobial prescribing associated with digital stewardship interventions. Automated prescribing warning systems, antibiotic order sets, and microbiology outcomes in electronic health record systems contribute in real-time to improved guideline adherence and length of therapy without adversely patient outcomes while minimizing broad-spectrum antibiotic use [162]. In hospital settings, integration of CDSS with microbiology and pharmacy databases has enabled previous de-escalation of therapy and enhanced adherence with stewardship recommendations [163].

AI-driven analytics further enhance stewardship efforts through predictive modelling of AMR trends, optimization of empiric therapy, and the initial identification of patients at high risk of infection with multidrug-resistant organisms. Machine-learning algorithms applied to data sources collected routinely have demonstrated potential to support real-time decision-making in intensive care and high-burden settings [164]. These technologies furthermore facilitate large-scale antimicrobial utilisation monitoring, allowing benchmarking of prescribing practices across organizations and regions [165].

Despite these auspicious and favorable developments, there are sizeable and ample problems to digital and AI-enabled stewardship implementation: the quality and quantity of data, data compatibility, approval among clinicians, and resource limitations, notably within the LMIC setting. To most effectively bridge the gap towards true integration of these technologies, a multidisciplinary approach should be applied, integrating user-centric system design with an approach profoundly rooted in present stewardship frameworks and clinical workflows [166]. Nonetheless, digital condition and AI represent crucial enablers of scalable, sustainable, and future-ready antimicrobial stewardship methods. The tools used in digital AMR and their impact are given in Table 1.

**Table 1:** Digital AMS Tools and Their Impact.

Digital AMS Tool	Description/Function	Impact on Antimicrobial Use	References
Electronic Health Records (EHRs)	Integration of prescribing, laboratory, and clinical data to support real-time monitoring of antimicrobial use	Enables audit and feedback, improves guideline adherence, and facilitates de-escalation	[167]
Clinical Decision Support Systems (CDSS)	Automated alerts, dosing guidance, drug–bug matching, and guideline-based recommendations	Reduces inappropriate broad-spectrum antibiotic use and duration of therapy	[168,169]
Electronic Prescribing Systems	Digital prescription platforms with stewardship rules and restriction policies	Decreases prescribing errors and non-indicated antimicrobial use	[170,171]
Rapid Diagnostic & Microbiology Integration	Real-time linkage of culture, susceptibility, and molecular diagnostics with prescribing systems	Enables early targeted therapy and timely de-escalation	[172]
AI-Based Predictive Analytics	Machine-learning models predicting resistance patterns and infection risk	Optimizes empiric therapy and supports resistance trend forecasting	[173]
Antimicrobial Utilization Dashboards	Visualization tools for tracking antimicrobial consumption and resistance trends	Supports benchmarking, surveillance, and institutional stewardship reporting	[174]
Mobile Health (mHealth) Applications	Smartphone-based stewardship tools for guideline access and decision support	Improves prescriber compliance, especially in resource-limited settings	[175]

### 7.5. Policy, Regulatory, and One Health Implications

Antimicrobial Resistance is increasingly recognised as a crucial global policy concern owing to its significant implications for public health, medical care, and socio-economic development. The World Health Organization (WHO) adopted the Global Action Plan on Antimicrobial Resistance (GAP-AMR) in 2015 to address the growing threat. It consists of five strategic objectives that aim to increase awareness, enhance monitoring, rationalize the use and development of antibiotics, and promote innovation and research [176]. The GAP-AMR highlights organized and synchronized action across human, animal, and environmental sectors, a fundamental rule of the One Health approach, to address the intricate, interconnected drivers of resistance [177].

The influential Review on Antimicrobial Resistance, led by R Price (2016), emphasized that antimicrobial resistance (AMR) poses as great a threat to global health in the 21st century as major challenges such as climate change and terrorism [178]. This document catalyzed national action plans in several countries, driving calls for legislative frameworks to control antibiotic prescribing, enhance stewardship programmes, and limit over-the-counter antimicrobial sales. It moreover underlined the condition for economic incentives to support new antibiotic development and innovation in diagnostics and therapeutics [179].

One Health is a significant and essential aspect of proper management of antimicrobial resistance because multidrug-resistant microorganisms and/or resistance genes move between human, animal, food, and environmental domains. There is evidence from a study indicating that antimicrobials used in agriculture can lead to the development of resistance gene sources, which are transferred from animals to human germs through direct contact or contamination of food and/or environments [180]. National policies that integrate monitoring data across sectors and enable joint stewardship interventions are more effective in curbing resistance than siloed methods [181,182].

Despite these worldwide standards, there are implementation problems in low- and middle-income countries (LMICs), including the absence of rule enforcement, limited diagnostic capacity, and underdeveloped systems of effective management. Multisectoral coordination, political commitment, funding, and capability building at the organizational level could ensure effective measurement of policy outputs at the worldwide level [183].

Antimicrobial resistance is a global health threat that extends far beyond microbiological failure, directly affecting patient survival and the sustainability of modern medicine. Addressing this difficulty necessitates a paradigm shift where antimicrobial effectiveness is prioritized alongside drug effectiveness and safety through integrated stewardship and personalized dosing [184].

Resistant infections create a notable strain on medical care systems and individual patient results:

- **Increased Mortality:** Patients with multidrug-resistant organism (MDRO) infections face a substantially greater risk of mortality, estimated at 1.7 times greater than those with open and exposed infections.
- **Morbidity and Readmissions:** Beyond first and beginning treatment failure, MDROs are associated with greater rates of hospital readmission, frequently doubling the probability of a patient returning within 30 days of discharge [185].
- **Healthcare Costs:** The need for more intense care, prolonged hospital stays, and the application of expensive, frequently toxic last-line medications drives up overall expenditures.
- **Therapeutic Risks and Inappropriate Exposure:** Inappropriate antimicrobial exposure, whether through erroneous dosing or prolonged duration, amplifies the risk of dose-dependent toxicities [186].
- **Common Toxicities:** Antimicrobials are regular and recurring causes of drug-induced liver injury (hepatotoxicity) and kidney injury (nephrotoxicity) [187].
- **Vulnerable Populations:** Toxicity risks are especially acute in vulnerable groups where individual pharmacokinetics differ considerably, making a “one-size-fits-all” dosing plan risky and hazardous [188].
- **Selective Pressure:** The overuse of broad-spectrum antibiotics, such as carbapenems, increases particular pressure, which accelerates the emergence of resistant bacteria and narrows future therapeutic choices [189].

As resistance to common antibiotics grows, clinicians increasingly rely on last-resort agents like **polymyxins** (colistin and polymyxin B).

- **Narrow Therapeutic Index:** While necessary and fundamental for treating multidrug-resistant Gram-negative germs, polymyxins are extremely nephrotoxic.
- **Emerging Alternatives:** To reduce reliance on these toxic agents, new β-lactam/-lactamase inhibitor combinations (like ceftazidime-avibactam) are being used as safer, more effective options for distinct resistant strains [190].

With the goal of optimizing outcomes through Stewardship and TDM in order to curb resistance and improve patient-level results, as shown in the Table 2, the sources highlight various vital and crucial plans [184].

## 8. Novel Therapies to Treat AMR

Recent advancements in therapies to combat antimicrobial resistance focus on innovative, non-traditional methods beyond standard antibiotics. Promising methods that expand the range of objectives and processes to overcome resistant pathogens [195,196]. Combination therapies integrating advanced treatments with natural substances, such as essential oils and propolis, are also being investigated to enhance effectiveness and reduce the development of resistance [197]. Cutting-edge genomic instruments such as CRISPR-Cas systems permit precise targeting of bacterial genomes to disrupt resistance genes, while nanotechnology and designed peptides offer new ways to disrupt biofilms and bacterial communication (quorum sensing) [198]. Immunotherapy, vaccines, and drug repurposing further contribute to a multi-pronged approach against AMR, targeting to improve treatment results and extend antibiotic lifespans [199]. The novel therapies used to treat antimicrobial resistance are listed in Table 3, along with their limitations.

## 9. Discussion

This review synthesizes global evidence on antimicrobial utilization and resistance across healthcare settings and populations, highlighting consistent patterns of high antimicrobial consumption, increasing resistance, and variability in stewardship implementation. However, interpretation of these findings requires careful consideration of methodological limitations and heterogeneity in the underlying evidence base.

A major limitation across included studies is the substantial variation in surveillance quality and data availability between regions. High-income countries benefit from well-established surveillance systems such as ESAC-Net and GLASS, enabling relatively robust longitudinal analyses and cross-country comparisons. In contrast, many low- and middle-income countries (LMICs) face fragmented surveillance infrastructure, limited microbiological capacity, and incomplete data coverage, particularly in primary care and community settings. As a result, global estimates of antimicrobial use and resistance are likely influenced by systematic underreporting and selection bias, with LMIC data often derived from tertiary centres that may not reflect broader population-level trends. In addition, the heterogeneity of included evidence represents an important constraint. This review integrates findings from diverse sources, including surveillance datasets, observational studies, and registry-based analyses, each with varying methodological rigor. Many studies are ecological in nature, limiting causal inference about the relationship between antimicrobial use and resistance. Furthermore, differences in study design, population characteristics, and outcome definitions reduce comparability and may contribute to inconsistencies in reported resistance patterns. The absence of standardized methodologies across regions further complicates synthesis and interpretation.

The use of aggregate consumption metrics, particularly the Defined Daily Dose (DDD), also introduces important limitations. While DDD is widely used for benchmarking antimicrobial use, it does not account for patient-level factors such as age, weight, renal function, or disease severity. Consequently, DDD may overestimate or underestimate actual antimicrobial exposure, especially in pediatric populations, intensive care settings, and contexts where dose adjustments are common. Reliance on single metrics without complementary measures—such as Days of Therapy (DOT) or spectrum-based indices—may therefore provide an incomplete representation of antimicrobial utilization.

Another critical consideration is the limited integration of antimicrobial use, resistance, and clinical outcomes at the patient level. Many surveillance systems capture consumption and resistance data independently, without linking them to clinical indications, treatment appropriateness, or patient outcomes. This restricts the ability to evaluate the effectiveness of stewardship interventions and to identify causal pathways between antimicrobial exposure and resistance development.

The review also adopts a One Health perspective, integrating evidence across human, animal, and environmental domains. While this approach reflects the interconnected nature of AMR, it introduces additional complexity. Data from these sectors are often collected using different methodologies, indicators, and reporting frameworks, limiting direct comparability. Moreover, evidence from animal and environmental studies is frequently less standardized and less systematically monitored than human healthcare data, particularly in LMICs. As a result, conclusions regarding cross-sectoral transmission of resistance should be interpreted with caution, and there remains a need for harmonized, integrated surveillance systems that enable meaningful cross-domain analysis.

Despite these limitations, several consistent findings emerge. Antimicrobial consumption remains highest in settings with limited regulatory control and high infectious disease burden, and is increasingly characterized by reliance on broad-spectrum and Watch-category antibiotics. Vulnerable populations experience disproportionate exposure and associated risks, including toxicity and treatment failure. Registry-based analyses consistently demonstrate associations between antimicrobial use and resistance trends, supporting the central role of stewardship interventions.

From a clinical and policy perspective, these findings reinforce the need for context-specific, data-driven antimicrobial stewardship strategies. However, future progress will depend on addressing current methodological gaps. Strengthening surveillance systems in LMICs, improving standardization of data collection, and integrating patient-level clinical information are essential priorities. In addition, combining multiple antimicrobial use metrics and expanding monitoring beyond hospital settings will provide a more accurate and comprehensive understanding of antimicrobial exposure.

Overall, while the evidence base is heterogeneous and subject to important limitations, the convergence of findings across diverse settings underscores the urgency of coordinated global action to optimize antimicrobial use and mitigate the progression of resistance.

## 10. Conclusions

Antimicrobial resistance (AMR) represents a rapidly escalating global public health challenge driven primarily by inappropriate and excessive antimicrobial use across human, animal, and environmental sectors. This review highlights substantial regional variation in resistance patterns, with particularly high rates reported in Southeast Asia and India. Increased reliance on Watch-category antibiotics and the widespread availability of over-the-counter antimicrobials further accelerate resistance, especially in low- and middle-income countries.

Vulnerable populations—including pediatric, elderly, pregnant, and immunocompromised individuals—are disproportionately affected, with higher risks of resistant infections and adverse drug-related outcomes. In addition to resistance, excessive antimicrobial exposure is associated with significant safety concerns, including nephrotoxicity, hepatotoxicity, and disruption of the microbiome.

Addressing AMR requires coordinated, multisectoral action through integrated One Health stewardship approaches. Strengthening surveillance systems, optimizing antimicrobial prescribing practices, improving regulatory control over antimicrobial distribution, and expanding access to rapid diagnostic tools are essential. Emerging strategies, including digital health technologies and clinical decision support systems, may further enhance stewardship efforts.

Overall, sustained, evidence-based interventions aligned across policy, clinical practice, and public health systems are critical to mitigate the global burden of AMR and preserve the effectiveness of existing antimicrobial therapies.

## 11. Future Perspectives

Future efforts to control antimicrobial resistance (AMR) should focus on evidence-informed, precision stewardship strategies that build on the patterns of antimicrobial use and resistance identified in this review. The integration of rapid molecular diagnostics and real-time surveillance systems is essential to guide targeted therapy, enable early de-escalation, and reduce unnecessary exposure to broad-spectrum and Watch-category antibiotics.

Digital health technologies, including clinical decision support systems (CDSS) and artificial intelligence (AI)-driven prescribing tools, offer promising approaches to improve adherence to treatment guidelines and optimize antimicrobial use in diverse healthcare settings. In parallel, strengthened regulatory frameworks, particularly in low- and middle-income countries, are required to limit non-prescription antibiotic use and improve access to diagnostic services.

Given the limited antibiotic development pipeline, adjunct and alternative strategies—such as bacteriophage therapy, antivirulence approaches, microbiome-preserving interventions, and host-directed therapies—require further clinical evaluation to establish their safety and effectiveness. Preventive strategies, including next-generation vaccines, may also reduce the burden of infection and subsequent antimicrobial demand, particularly in high-risk populations.

Sustained progress against AMR will depend on the implementation of coordinated One Health approaches that integrate surveillance and stewardship across human, animal, and environmental sectors. Future research should prioritize translational studies that bridge emerging technologies with clinical practice to ensure scalable and context-specific solutions.

## Figures and Tables

**Table 2:** Different Strategy for Antimicrobial Resistance and Clinical Impact.

Strategy	Objective	Clinical Impact	References
AMS Programs	Optimize drug selection, dose, and duration.	Lower mortality, shorter stays, and cost savings	[191]
Rapid Diagnostics	Identify pathogens and susceptibility quickly.	Reduces unnecessary use of broad-spectrum empirical therapy.	[192]
Antibiotic De-escalation	Transition from broad-spectrum to narrow-spectrum drugs once results are known.	Minimizes the risk of resistance development and unnecessary drug exposure.	[193]
Therapeutic Drug Monitoring (TDM)	Measure blood concentrations to guide individualized dosing.	Proactively prevents toxicity while ensuring effective drug levels.	[194]

**Table 3:** Novel Therapy Used in Antimicrobial Resistance.

Sr. No	Novel Therapy	Use	Limitations	References
1	Bacteriophages and phage-derived enzymes (endolysins)	High specificity for targeting germs, active against multidrug-resistant strains, can penetrate biofilms when designed or combined with enzymes.	Narrow host range immune clearance, regulatory and production difficulties.	[200]
2	Antimicrobial peptides	Broad-spectrum activity, fast bactericidal action, activity vs. biofilms and some MDR pathogens.	Hemolytic/toxic effects at high doses, proteolytic deterioration.	[201,202]
3	CRISPR-Cas established antimicrobials	Removing resistance plasmids or killing solely resistant bacteria enables precision microbiome editing.	Safe delivery in vivo remains an important and significant hurdle due to potential off-target effects and immune responses.	[203]
4	Antibody–antibiotics conjugates (AACs)	Improves local drug concentration, reduces systemic exposure and toxicity, reach intracellular pathogens.	Complex production, cost, and conditions for good bacterial surface goals.	[204]
5	Microbiome and bacteriotherapy techniques	Prevents infection and colonization, can reduce antibiotic application and choice pressure.	Variable effectiveness, regulatory intricacy, donor screening and safety concerns for FMT.	[205]
6	Anti-virulence and quorum-sensing inhibitors	Less selection pressure for resistance because these agents disarm rather than kill bacteria; good as adjunctive therapies.	Often pathogen and mechanism-specific, effectiveness may depend on host immune competence.	[206,207]
7	Nanoparticles, liposomal delivery	Improve PK/PD, overcome permeability obstacles, reduce systemic toxicity, and can synergize with existing antibiotics.	Safety/toxicity and scale-up/regulatory limitations continue.	[207,208]
8	Phytochemicals and herb-based antimicrobials	Disrupt bacterial membranes, inhibit efflux pumps, and interfere with resistance enzymes, frequently showing synergistic effects when combined with existing antibiotics	Variability in phytochemical composition, absence of uniform dosing, potential toxicity, and inadequate clinical trials to validate safety and effectiveness	[209]

## Data Availability

This is a review article, and no new data were generated or analysed in this study. All the data discussed in this review article have been referenced to their original sources.

## Abbreviations

The following abbreviations are used in this manuscript:

AMR	Antimicrobial Resistance
AMU	Antimicrobial Use
WHO	World Health Organization
AWaRe	Access, Watch, Reserve (classification)
PKs	Pharmacokinetics
PDs	Pharmacodynamics
SSA	Sub-Saharan Africa
MRSA	Methicillin-Resistant *Staphylococcus aureus*
ESBL	Extended-Spectrum Beta-Lactamase
GLASS	Global Antimicrobial Resistance and Use Surveillance System
LMIC	Low- and Middle-Income Countries
AMS	Antimicrobial Stewardship
PHC	Primary Health Care
ASP	Antimicrobial Stewardship Program
DOT	Days of Therapy
ICU	Intensive Care Unit
OTC	Over-the-Counter
MDR	Multidrug Resistance
AKI	Acute Kidney Injury
HIV	Human Immunodeficiency Virus
OPD	Outpatient Department
ADR	Adverse Drug Reactions
ED	Emergency Department
ESAC-Net	European Surveillance of Antimicrobial Consumption Network
ECDC	European Centre for Disease Prevention and Control
EARS-Net	European Antimicrobial Resistance Surveillance Network
DDD	Defined Daily Dose
DASC	Days of Antibiotic Spectrum Coverage
ASI	Antibiotic Spectrum Index
CDSS	Clinical Decision Support Systems
AI	Artificial Intelligence
EHR	Electronic Health Record
GAP-AMR	Global Action Plan on Antimicrobial Resistance
FMT	Fecal Microbiota Transplantation
AACs	Antibody–Antibiotic Conjugates
